# GPR84 and Neuroinflammation: A Receptor Worth Targeting, or A Target Worth Reconsidering?

**DOI:** 10.1007/s12035-026-06132-z

**Published:** 2026-08-13

**Authors:** Julia Jarco, Natalia Malek

**Affiliations:** Department of Chemical Biology and Bioimaging, Wroclaw University of Science and Technology, Wroclaw, Poland

**Keywords:** GPR84, Neuroinflammation, Microglia, Neutrophils, Biased agonism, Neuropathic pain, TBI, Innate immunity

## Abstract

GPR84 is a medium-chain fatty acid (MCFA)-sensing G-protein-coupled receptor conditionally expressed on innate immune cells – neutrophils, monocytes, macrophages, and microglia – with expression strongly induced by inflammatory stimuli. Upon activation, GPR84 engages Gi/o signalling, MAPK/ERK, NF-κB, and NLRP3 inflammasome pathways, amplifying pro-inflammatory effector functions across peripheral and central myeloid populations. In microglia, receptor upregulation marks a disease-associated subpopulation linked to TNF-α and IL-1β production in models of neurodegeneration, traumatic CNS injury, and neuropathic pain. Yet the same receptor exhibits context-dependent protective roles: GPR84 deletion attenuates neuropathic hypersensitivity through peripheral macrophage reprogramming while leaving microglial activation intact, and Gpr84-deficient Alzheimer's disease mice show reduced microgliosis but paradoxically accelerated cognitive decline, demonstrating that the receptor's output is determined by cell type, disease stage, and local inflammatory context rather than by a fixed pro-inflammatory identity. Recent cryo-EM structural resolution of GPR84 has enabled the design of G-protein-biased agonists that preferentially engage pathways linked to motility and phagocytosis while sparing cytokine amplification, providing a rationale for selectively enhancing homeostatic clearance without exacerbating chronic inflammation. However, the most clinically advanced antagonist, GLPG1205, failed to meet primary endpoints in Phase II trials in ulcerative colitis and idiopathic pulmonary fibrosis. This review argues that these translational failures reflect deeper methodological limitations – including supraphysiological agonist concentrations, constitutive knockout models that conflate microglial and peripheral macrophage contributions, species selectivity gaps in available antagonists – that have produced conflicting preclinical evidence and impeded clinical translation. Resolving GPR84's context-dependent biology will require conditional genetic models, CNS-penetrant pharmacological tools, and integration of spatial and temporal multiomics approaches before the receptor's therapeutic potential can be meaningfully evaluated.

**Graphical Abstract:**

**Figure:**
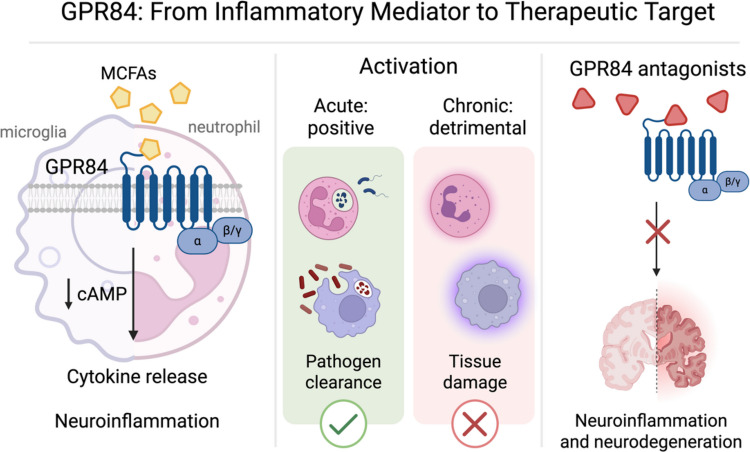

## Introduction

G-protein-coupled receptors (GPCRs) account for over one-third of approved drugs and represent the most therapeutically exploited membrane receptor family. Within this family, GPR84 has emerged as an immunometabolic receptor linking lipid sensing to innate immune activation. GPR84 is now established as a medium-chain fatty acid (MCFA) receptor selectively activated by C9–C14 fatty acids [1–4].

GPR84 is conditionally expressed on innate immune cells—predominantly neutrophils, monocytes, macrophages, and microglia—with expression strongly induced by inflammatory stimuli including lipopolysaccharide (LPS), TNF-α, and IFN-γ. Its activation triggers canonical Gαi/o-mediated signalling, MAPK cascades, NF-κB activation, and NLRP3 inflammasome engagement, collectively amplifying pro-inflammatory responses. Yet emerging evidence reveals a context-dependent, sometimes protective function, positioning GPR84 as a regulatory node—a double-edged sword—in chronic inflammation and neurodegeneration. This review synthesizes current knowledge of GPR84 molecular pharmacology, cellular biology, and therapeutic relevance, with emphasis on its dichotomous roles in neutrophils and microglia, the pharmacological landscape of biased agonism, and the translational lessons from clinical-stage antagonist development [1, 4–7].

## Molecular and Structural Characteristics of GPR84

GPR84 is a class A GPCR that plays a pro-inflammatory role in various physiological and pathological contexts. GPR84 belongs to the rhodopsin-like family of GPCRs and exhibits the canonical seven-transmembrane (7TM) helical structure, with an extracellular N-terminus and an intracellular C-terminus. Two research groups first identified it in 2001 through transcriptomic screening studies conducted independently [8, 9]. In humans, the GPR84 gene is located on the minus strand of chromosome 12 and comprises noncoding intronic sequences that give rise to at least two transcript variants. The major isoforms, GPR84-201 and GPR84-202, both encode proteins consisting of 396 amino acids, with differences in exon organization. These isoforms are predicted to function similarly and translate into integral membrane proteins with a molecular mass of approximately 43.5 kDa [10].

The 2023 cryo-electron microscopy (cryo-EM) structure of GPR84 in complex with the synthetic agonist 6-OAU and a Gαi heterotrimer has provided the first atomic-resolution insight into ligand recognition and receptor activation [11]. The orthosteric binding pocket is located within the transmembrane bundle, with key hydrophobic residues in TM3, TM5, and TM6 forming close contacts with the acyl chain of the fatty acid ligand. Chain-length selectivity is determined by the depth and hydrophobicity of this pocket: medium-chain fatty acids (C9–C14) optimally fill the binding cavity, while shorter or longer chains display progressively reduced potency. The amine group of 6-OAU forms a hydrogen bond with asparagine residues at the top of the binding site, and this interaction is critical for high-potency engagement. Receptor activation induces the canonical TM6 outward movement associated with class A GPCR activation, creating an intracellular cavity that accommodates the Gαi subunit. These structural insights directly inform the rational design of biased ligands: subtle modifications to the ligand scaffold can shift receptor conformations toward G-protein-selective or β-arrestin-selective states, providing a molecular basis for pathway-selective therapeutic targeting [11].

GPR84 shares approximately 20–30% sequence homology with related free fatty acid receptors and is most closely related to GPR120. The extracellular loops, particularly ECL2, contribute significantly to binding pocket architecture and help define the selectivity filter for medium-chain lipid ligands. The second extracellular loop adopts a β-hairpin conformation that caps the binding pocket from above, establishing additional contact points with ligand head groups. Mutagenesis studies conducted prior to the cryo-EM structure had identified residues including Arg172, Tyr244, and His103 as important for ligand binding and receptor activation; these residues are broadly consistent with the contacts observed in the solved structure. The intracellular surface, particularly ICL3 and the cytoplasmic tail, provides the coupling interface for heterotrimeric G-proteins, with selectivity for the Gi/o family encoded by specific ICL2 and ICL3 residue contacts [6, 11].

### GPR84-dependent Signalling Pathways

**Fig. 1:**
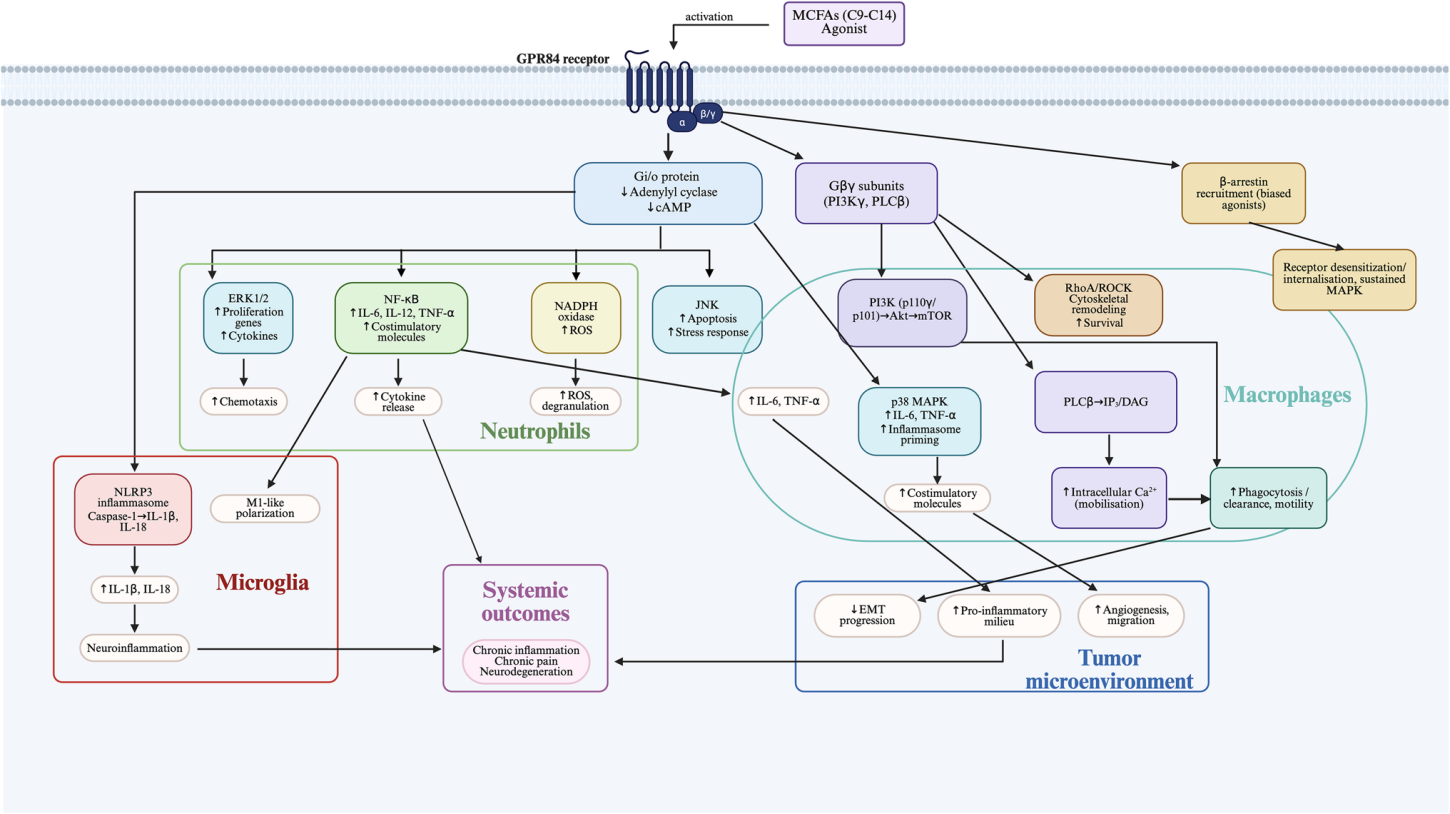
GPR84-dependent signalling pathways and potential axes of ligand-dependent bias. Upon stimulation by medium-chain fatty acids (MCFAs, C9–C14) or synthetic agonists, GPR84 couples to Gi/o proteins, leading to inhibition of adenylate cyclase and reduced intracellular cAMP levels. This activation triggers multiple cascades, including MAPKs (ERK1/2, JNK, p38), PI3K–Akt–mTOR, NF-κB, ROS production via NADPH oxidase, RhoA/ROCK-mediated cytoskeletal remodelling, and NLRP3 inflammasome activation. These pathways mediate cell-specific responses: in neutrophils (chemotaxis, ROS release, degranulation), in microglia (M1-like polarization, inflammasome priming, neuroinflammation), and in macrophages (IL-6, TNFα secretion, costimulatory signalling). These effects promote systemic outcomes such as chronic inflammation, neurodegeneration, and chronic pain. Ligand-dependent signalling bias is indicated schematically by differential weighting of the Gi/Gβγ versus β-arrestin branches. Whereas the orthosteric agonists 6-OAU and 2-HTP promote robust GPR84–arrestin-3 recruitment, the G-protein-biased agonist DL-175 preferentially engages Gi/Gβγ-dominated pathways (cAMP suppression, PI3Kγ–PLCβ activation, Ca.^2+^ mobilization, chemotaxis and phagocytosis) with markedly reduced β-arrestin recruitment and delayed Akt activation. Newer, highly G-protein-biased agonists such as OX04528 and OX04529 display picomolar cAMP potency with no detectable β-arrestin recruitment, illustrating that the Gi/Gβγ and β-arrestin outputs of GPR84 can be pharmacologically separated. This conceptual framework provides a mechanistic rationale for designing GPR84 modulators that spare or enhance homeostatic phagocytic outputs while selectively limiting NF-κB- and NLRP3-driven cytokine amplification in chronic inflammatory and fibrotic conditions [3, 26, 51]

The major GPR84-dependent signalling pathways—including Gi/o coupling, MAPK (ERK1/2 and p38), PI3K/Akt activation, NF-κB signalling, and NLRP3 inflammasome engagement—are summarized in Fig. 1. These pathways collectively mediate classical innate immune effector functions, metabolic reprogramming, and inflammasome priming across neutrophils, macrophages, and microglia [7, 11–15].

### Classical Gi/o Signalling

The best-characterized mechanism of GPR84 activity involves coupling to Gi/o proteins. Upon ligand engagement, conformational changes in the receptor promote interaction with heterotrimeric Gi/o complexes, leading to dissociation of the Gα and Gβγ subunits. The Gαi subunit directly inhibits adenylyl cyclase, lowering intracellular cAMP concentrations. This reduction in cAMP reduces PKA activity and downstream phosphorylation events, effectively lowering the threshold for cellular activation. In innate immune cells, this primes cells for enhanced responsiveness to inflammatory stimuli. In neutrophils, suppressed cAMP levels enhance responsiveness to chemoattractants, increase adhesion through integrin upregulation, and potentiate the oxidative burst mediated by NADPH oxidase. In macrophages, Gi/o signalling promotes metabolic reprogramming toward glycolysis and supports transcriptional activation of pro-inflammatory genes. In microglia, diminished cAMP favours a shift toward pro-inflammatory phenotypes, contributing to enhanced NLRP3 inflammasome priming and cytokine release. Thus, Gi/o coupling provides the core pro-inflammatory output of GPR84, linking receptor stimulation directly to acute effector functions [4, 13, 14, 16].

### Gβγ-mediated Signalling: PI3K, PLCβ, and Cytoskeletal Control

Beyond the classical Gαi-mediated inhibition of adenylyl cyclase, dissociation of the GPR84-coupled Gi heterotrimer also liberates free Gβγ dimers that engage a distinct set of effectors. In innate immune cells, Gβγ complexes are direct upstream activators of the p101/p110γ and p84/p110γ PI3Kγ complexes and of PLCβ, thereby linking Gi-coupled receptors to the pathways that govern chemotaxis, intracellular Ca^2+^ mobilization, and cytoskeletal remodelling. PI3Kγ-dependent generation of phosphatidylinositol-3,4,5-triphosphate at the leading-edge coordinates front-rear polarity and directional migration in neutrophils and monocytes, and Gβγ release is sufficient to direct neutrophil chemotaxis independently of receptor or Gα activation. In parallel, Gβγ-activated PLCβ drives a PKCβ/PKD and slingshot-cofilin axis that controls actin depolymerisation and directional motility during GPCR-mediated chemotaxis. Because GPR84 agonists such as 6-OAU and embelin elicit precisely these outputs—robust chemotaxis, adhesion-molecule upregulation, membrane ruffling, and actin-dependent cytoskeletal rearrangement in neutrophils, macrophages, and microglia—a balanced treatment of Gαi- and Gβγ-driven signalling is warranted, with the Gβγ-PI3Kγ-PLCβ arm emerging as a central determinant of GPR84-dependent motility and phagocytic competence [17–20].

### Intracellular Ca^2+^ Signalling

Intracellular Ca^2+^ mobilization is a consistent and functionally important feature of GPR84 activation, consistent with engagement of the Gβγ-PLCβ arm described above. Indeed, GPR84 is highly amenable to Ca^2+^-flux-based assays: high-throughput screening of 160,000 compounds using a Ca^2+^ mobilization readout in HEK293 cells co-expressing GPR84 and Gα16 identified the potent, selective agonist 2-(hexylthio)pyrimidine-4,6-diol (ZQ-16), which triggers robust Ca^2+^ transients alongside inhibition of cAMP accumulation, ERK1/2 phosphorylation, and receptor internalization [21]. The same platform has been widely used to characterize both GPR84 agonists and antagonists, confirming that the receptor couples efficiently to intracellular Ca^2+^ release. Because these Ca^2+^ signals are tightly linked to actin remodelling, respiratory burst, and phagocytosis in myeloid cells, Ca^2+^ should be regarded as a key second messenger coupling GPR84 activation to rapid innate effector functions, and any mechanistic model of GPR84 signalling should incorporate the Gβγ-PLCβ-Ca^2+^ axis alongside Gαi-mediated cAMP suppression [4].

### MAPK and Kinase Cascades: ERK, PI3K/Akt, and p38 MAPK

Activation of GPR84 through Gi/o coupling initializes multiple downstream kinase cascades that extend its influence beyond cAMP suppression. One of the earliest and most consistent signals observed is the phosphorylation of ERK1/2 [13]. This pathway regulates cytoskeletal remodelling and motility, enabling neutrophils and macrophages to migrate efficiently toward chemotactic gradients. In neutrophils, ERK activation drives integrin upregulation (CD11b/CD18), firm adhesion to the endothelium, and transmigration into inflamed tissues. In macrophages and monocytes, ERK promotes pro-inflammatory polarization and enhances transcription of cytokines such as TNF-α and IL-6, amplifying immune responses [4, 14]. In microglia, ERK signalling contributes to morphological changes including process extension and membrane ruffling, as well as to inflammatory mediator release [6].

Parallel to ERK, GPR84 stimulates the PI3K/Akt pathway, which integrates metabolic signals with cell survival and inflammatory activity. PI3K activation generates phosphatidylinositol (3,4,5)-trisphosphate, recruiting and activating Akt. This leads to enhanced glycolytic flux, increased resistance to apoptosis, and potentiation of NF-κB signalling. In macrophages, PI3K/Akt signalling supports inflammasome priming and sustained cytokine production, while in microglia it reinforces a shift toward pro-inflammatory phenotypes and promotes IL-1β secretion. PI3K/Akt is also implicated in the regulation of phagocytic capacity, highlighting its importance in innate immune effector functions [14, 16, 22].

A third major signalling axis downstream of GPR84 is p38 MAPK. This stress-responsive kinase integrates inflammatory cues and receptor activation to promote chemokine and cytokine production. In neutrophils, p38 enhances chemotaxis and potentiates NADPH oxidase-dependent oxidative burst. In macrophages, p38 contributes to the secretion of IL-8 and other mediators that recruit additional immune cells to sites of inflammation. In microglia, p38 activation amplifies reactive oxygen species production and has been linked to neuronal injury in models of neuroinflammation [6, 14, 16, 22].

### Alternative signalling: β-arrestin, NF-κB, and Inflammasome Pathways

Beyond classical Gαi/o coupling and MAPK activation, GPR84 may also engage in alternative signalling routes that diversify its functional output. Recruitment of β-arrestins, although variable depending on ligand scaffold, represents a critical branch point in receptor biology. β-arrestin binding not only mediates receptor desensitization and internalization, but also scaffolds signalling complexes that activate ERK, JNK, or p38 from endosomal compartments, generating signals that are temporally prolonged and spatially restricted compared to G-protein outputs. This property allows β-arrestin–biased ligands to fine-tune immune cell activity, attenuating overactivation while maintaining basal responsiveness [5].

Another major axis of GPR84-dependent signalling involves NF-κB, a master regulator of immune gene expression. Crosstalk between GPR84 and Toll-like receptors produces strong synergistic activation of NF-κB, markedly increasing transcription of TNF-α, IL-6, and other pro-inflammatory mediators. In macrophages, this synergy amplifies classically activated macrophage phenotype, while in neutrophils it enhances chemokine production that recruits additional leukocytes. In the CNS, NF-κB activation downstream of GPR84 contributes to sustained microglial reactivity, creating a feed-forward loop of cytokine release and neuronal stress [13, 22, 23]. These observations support the view that GPR84 functions predominantly as an “inflammation amplifier” rather than a primary initiator of innate immune activation. In macrophages and microglia, GPR84 stimulation alone generally fails to elicit high-level cytokine production but potently boosts NF-κβ activation, pro-inflammatory mediator expression, and NLRP3 inflammasome signalling when Toll-like receptors or other pattern-recognition receptors are co-engaged. In human neutrophils, GPR84 activation on its own triggers only a modest oxidative activity once the formyl-peptide receptors have been desensitized, again consistent with a modulatory, hierarchy-dependent role rather than a canonical danger sensor acting in isolation. [14, 23, 24].

Perhaps the most pathologically relevant output of GPR84 stimulation is its influence on the NLRP3 inflammasome. By lowering cAMP and simultaneously activating ERK, PI3K/Akt, and NF-κB, GPR84 primes microglia and macrophages for inflammasome assembly. Experimental studies have shown that stimulation with synthetic agonists enhances caspase-1 activation, IL-1β release, and pyroptotic cell death, all hallmarks of inflammasome engagement. In disease models, this has been linked to synaptic pruning, oxidative stress, and neurotoxicity, implicating GPR84 as a driver of chronic neuroinflammation [12, 16, 23, 25].

### Signalling Bias as an Emerging Layer of GPR84 Regulation

Importantly, accumulating pharmacological evidence indicates that GPR84 signalling is highly ligand-dependent and cannot be assumed to uniformly engage all downstream pathways described above. Recent structure–activity studies have demonstrated that certain synthetic agonists selectively activate G_i_-mediated inhibition of cAMP while showing little or no β-arrestin recruitment, establishing GPR84 as a receptor amenable to functional selectivity. DL175 and its optimized derivatives OX04528 and OX04529 exhibit picomolar potency in Gi signalling yet fail to induce detectable β-arrestin engagement, highlighting a strong bias toward G-protein pathways. These findings suggest that activation of MAPK, NF-κB, or inflammasome-associated responses is likely influenced by ligand-specific receptor conformations rather than representing obligatory outputs of GPR84 activation. Signalling bias introduces an additional regulatory layer that critically shapes the inflammatory consequences of GPR84 activation and provides a mechanistic basis for pathway-selective therapeutic targeting [26]. Therapeutically, Gi-biased agonists suit acute immune activation (infection, immunotherapy), while β-arrestin-favouring or antagonistic ligands (e.g., GLPG1205) target chronic inflammation by promoting desensitization. CNS effects remain context-dependent, requiring tailored modulators [5, 12].

### Endogenous and Synthetic Ligands of GPR84

GPR84 is primarily activated by medium-chain fatty acids (MCFAs), ranging in length from 9 to 14 carbon atoms, with capric acid (C10), undecanoic acid (C11), and lauric acid (C12) being the most effective. These lipids represent context-dependent metabolic cues whose availability and physiological impact are tightly linked to nutritional state, inflammatory signals, and microbial activity [10, 14].

The case for MCFAs as physiological ligands is genuinely weak. Circulating fatty acids in plasma reach concentrations in the low micromolar range under most conditions, yet the in vitro EC₅₀ values for MCFAs at GPR84 are typically higher in any functional cellular readouts. The gap widens further in the CNS, where MCFAs cross the blood–brain barrier poorly and where their local concentrations in the parenchyma are largely unmeasured. This leaves GPR84 functionally orphaned in the sense that its tonic in vivo activation state remains unknown.

A study by Cox et al. demonstrated that age-associated gut dysbiosis, particularly expansion of *Parabacteroides goldsteinii*, elevates circulating MCFAs and 3-hydroxyoctanoic acid that activate GPR84 on peripheral myeloid cells, triggering IL-1β/TNF-α release, vagal afferent impairment, and hippocampal memory deficits, establishing the gut microbiome as a disease-state-dependent endogenous ligand reservoir for GPR84 with direct CNS consequences [27]. This axis is particularly relevant to neurological diseases characterised by dysbiosis, including Alzheimer's, where chronically elevated microbial MCFAs may prime peripheral myeloid cells and amplify neuroinflammation.

**Fig. 2:**
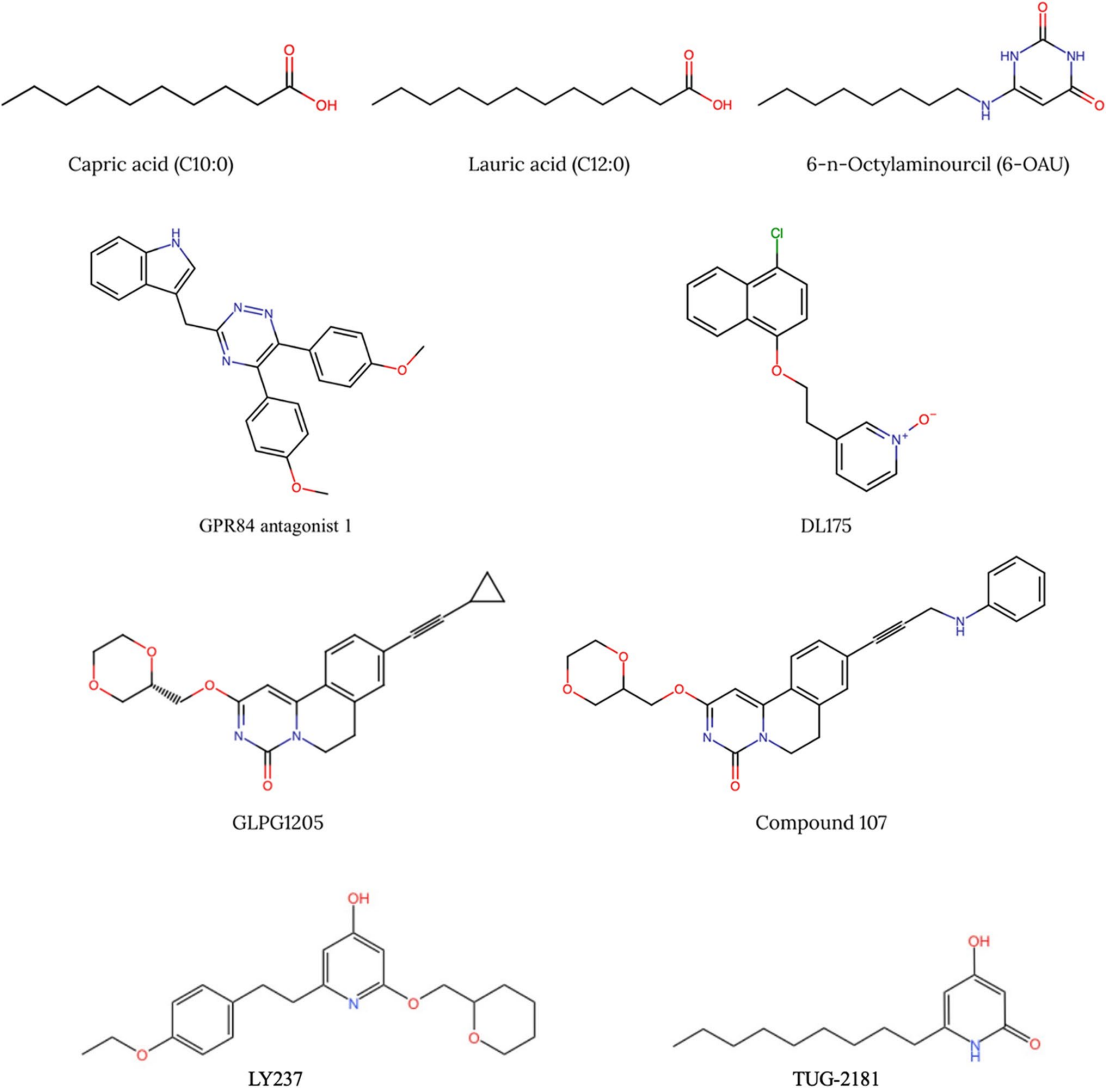
Representative chemical structures of GPR84 ligands. Chemical structures of representative GPR84 ligands. Endogenous agonists: capric acid (C10:0) and lauric acid (C12:0); synthetic agonists: 6-n-octylaminouracil (6-OAU), DL175 and LY237 (6-nonylpyridine-2,4-diol), a highly potent lipid-mimetic GPR84 agonist; selective antagonists: GPR84 antagonist 1, GLPG1205, Compound 107 and TUG-2181, a low-nanomolar inverse agonist/antagonist that fully inhibits GPR84-mediated neutrophil activation. Structures were redrawn using ChemSketch based on published data

The limited potency and lack of selectivity of endogenous ligands have driven the development of synthetic agonists as indispensable tools for dissecting GPR84 pharmacology. Representative structures of endogenous agonists (capric acid, lauric acid), synthetic agonists (6-OAU, DL175), and antagonists (GPR84 antagonist 1, GLPG1205, Compound 107) are shown in Fig. 2 [28–30]. Among the earliest and most extensively characterized compounds is 6-n-octylaminouracil (6-OAU), an uracil-derived small molecule that functions as a potent orthosteric agonist. 6-OAU robustly activates GPR84 in multiple cellular systems, producing strong inhibition of cAMP accumulation together with downstream signalling events including ERK phosphorylation and calcium mobilization. Functionally, these pathways translate into enhanced chemotaxis, upregulation of adhesion molecules, and increased production of reactive oxygen species in myeloid cells. Experimental studies in human neutrophils demonstrated that 6-OAU promotes robust CD11b expression and oxidative burst, effects that are abrogated by selective antagonists such as GPR84 antagonist 1, confirming receptor specificity. Owing to this reproducible pharmacological profile, 6-OAU has become a benchmark agonist for in vitro studies and experimental models of inflammation [4, 14].

Building on this foundation, medicinal chemistry efforts have expanded the structural space of GPR84 agonists. A major advance is DL175, another uracil-based molecule characterized as a G protein–biased agonist. Compared with 6-OAU, DL175 displays enhanced potency and preferential activation of Gi-mediated signalling, resulting in pronounced suppression of cAMP with minimal β-arrestin recruitment. Structure–activity studies further demonstrated that DL175 and its optimized derivatives (OX04528 and OX04529) robustly engage G-protein pathways while failing to elicit detectable arrestin signalling. Functionally, biased activation of GPR84 by DL175 promotes chemotaxis, adhesion, and oxidative burst in myeloid cells, establishing this compound as a valuable pharmacological probe for dissecting pathway-selective GPR84 signalling and modelling inflammatory responses driven by innate immunity [26, 29].

Beyond uracil derivatives, structurally distinct scaffolds have been developed to refine GPR84 pharmacology and improve drug-like properties. Compounds such as 2-(hexylthio)pyrimidine-4,6-diol and 6-nonylpyridone-2,4-diol retain nanomolar to sub-micromolar potency while exhibiting enhanced metabolic stability and oral bioavailability. These molecules expand the chemical diversity of GPR84 agonists and facilitate their application in animal models of inflammatory disease. Indeed, pharmacological activation of GPR84 using non-uracil agonists has been explored in fibrotic and intestinal inflammation models, highlighting the receptor’s contribution to myeloid-driven pathology across multiple organs [6, 31].

The most clinically advanced GPR84 antagonist to date is GLPG1205, an orally available small molecule developed by Galapagos NV that has progressed to Phase II clinical evaluation in idiopathic pulmonary fibrosis and ulcerative colitis. Preclinical studies demonstrate that GLPG1205 attenuates inflammatory features in experimental disease models, supporting its therapeutic potential in chronic inflammatory settings. Clinical development has further established its safety and pharmacodynamic activity in humans. While genetic and experimental evidence implicates GPR84 in microglial activation and neuroinflammatory processes, pharmacological studies with GLPG1205 have thus far primarily focused on peripheral inflammatory indications. These findings position GPR84 antagonism as a promising strategy for modulating maladaptive innate immune activation, although the full therapeutic scope of GLPG1205 continues to be defined [12, 32, 33].

Beyond GLPG1205, medicinal chemistry efforts have yielded structurally distinct antagonists such as Compound 107, a pyridone-based molecule characterized by high selectivity, nanomolar potency, and improved metabolic stability. Preclinical studies demonstrate that Compound 107 suppresses inflammatory gene expression and attenuates pathological features in experimental models of intestinal and pulmonary inflammation. These findings highlight the potential of next-generation GPR84 antagonists to extend therapeutic applications beyond respiratory disease to broader gastrointestinal and systemic inflammatory contexts [6, 28].

Notably, minimal structural modification of a GPR84 full agonist LY237 scaffold is sufficient to produce a functional switch to inverse agonism, yielding low-nanomolar compounds such as TUG-2181 that suppress constitutive receptor activity and fully inhibit GPR84-mediated neutrophil activation — indicating that GPR84 maintains ligand-independent basal signalling and that the boundary between agonism and inverse agonism is pharmacologically accessible with modest chemical changes [34].

Another improvement was made recently in the field, as a novel chromenopyrrole scaffold was identified and optimized as orthosteric GPR84 antagonists, with improved drug-like physicochemical properties relative to earlier tricyclic antagonist series, expanding the chemical toolkit available for selective GPR84 inhibition [35].

### GPR84 in Neutrophil Biology

Neutrophils serve as first responders of the innate immune system and are rapidly recruited to sites of infection or tissue injury. Under basal conditions, GPR84 expression in neutrophils is low; however, inflammatory stimuli such as lipopolysaccharide (LPS), TNF-α, or IFN-γ induce marked upregulation of receptor expression. Once expressed, GPR84 shapes both neutrophil migration and effector functions, thereby amplifying innate immune responses [14, 15].

In neutrophils, GPR84 activation amplifies classical neutrophil effector functions through signalling pathways described above. Functionally, this results in enhanced microbial killing but also promotes collateral tissue damage when activation is sustained [4]. Experimental data support these functions. Forsman et al. [14] demonstrated that GPR84 stimulation in human neutrophils increased CD11b expression, enhanced reactive oxygen species production, and promoted chemotaxis toward fMLP or IL-8, effects that were abrogated by the GPR84 antagonist 1. These responses were dependent on actin polymerization and ERK1/2 activity, underscoring the role of GPR84 in priming neutrophils for rapid and robust activation [14]. Beyond peripheral tissues, neutrophils are increasingly recognized as contributors to central nervous system pathology. Although normally excluded by the blood–brain barrier (BBB), neutrophils infiltrate the CNS during ischaemic stroke, multiple sclerosis, and systemic inflammation, where they promote barrier disruption and oxidative tissue injury. In experimental models of neuroinflammation, neutrophils mediate blood–spinal cord barrier breakdown through integrin-dependent adhesion and ROS production. While GPR84 is primarily characterized in peripheral innate immunity and is upregulated in neuroinflammatory settings, direct evidence linking GPR84-expressing neutrophils to BBB disruption or neuronal injury remains limited. Nevertheless, given the established role of neutrophils in CNS damage and the ability of GPR84 to amplify neutrophil activation, this receptor may represent an additional modulatory layer in neuroinflammatory disease [13, 36, 37].

GPR84 signalling also extends neutrophil activity through cooperation with formyl peptide receptors (FPR1/FPR2), prolonging reactive oxygen species generation beyond the desensitization phase of fMLP stimulation. This sustained activation is beneficial during acute infection but becomes detrimental when persistent, contributing to chronic inflammatory pathology.

### GPR84 in Macrophages and Microglia

Microglia are the resident macrophages of the central nervous system (CNS), responsible for synaptic pruning, tissue surveillance, and the orchestration of immune responses. Under homeostatic conditions, Gpr84 expression in microglia is low. In contrast, during inflammation, trauma, or neurodegeneration, its expression rises sharply, placing Gpr84 at the centre of disease-associated microglial states [13].

Transcriptomic and functional studies indicate that GPR84 expression is markedly upregulated in microglia under inflammatory conditions. Early work demonstrated increased receptor expression in neuroinflammatory models, while more recent studies have linked GPR84 activation to enhanced pro-inflammatory signalling and inflammasome engagement. In both macrophages and microglia, GPR84 activation has been consistently associated with inflammasome-driven inflammatory responses, while its inhibition attenuates these responses, supporting a role for the receptor as a modulator of innate inflammatory signalling [32]. These findings are supported by experimental studies demonstrating that GPR84 inhibition reduces inflammasome activation and IL-1β release in neuroinflammatory models, reinforcing its role in shaping microglial inflammatory responses [16].

Importantly, GPR84 does not act in isolation. Microglia co-express multiple Toll-like receptors (TLRs), and co-stimulation with TLR ligands and GPR84 agonists results in synergistic NF-κB activation, increased chemokine release (CCL2, CXCL10), and enhanced recruitment of peripheral macrophages. In models of neuropathic pain and TBI, GPR84 expression peaked alongside microglial activation and monocyte infiltration. Gpr84 knockout mice exhibited attenuated pain hypersensitivity, reduced pro-inflammatory gene expression, and diminished infiltration of CD68⁺ macrophages, underscoring the receptor’s role in coordinating both resident microglia and infiltrating myeloid cells [13, 16, 25, 38].

### GPR84 in T Lymphocytes and Adaptive Immunity

Although GPR84 is most prominently characterized in innate immune cells, it is also expressed at lower levels on T lymphocytes, particularly under inflammatory conditions. The earliest direct evidence came from Venkataraman & Kuo [39], who showed that GPR84 is highly expressed in splenic T and B cells, and that GPR84-deficient mice exhibit selectively increased IL-4 production following anti-CD3 stimulation,in vitro–differentiated Th2 cells from GPR84-deficient mice also produced higher levels of IL-4, IL-5, and IL-13, establishing GPR84 as a negative regulator of early IL-4 gene expression in activated T cells [39]. Functional studies suggest that GPR84 signalling in antigen-presenting cells influences the cytokine environment that shapes T helper cell polarization. GPR84 activation in macrophages and dendritic cells promotes secretion of IL-12, IL-23, and TNF-α, cytokines that favour Th1 and Th17 differentiation [40]. Consistent with this, GPR84 activation has been associated with enhanced IFN-γ and IL-17 production in CD4+ T cell populations in inflammatory contexts [22]. However, direct mechanistic evidence linking GPR84-intrinsic signalling in T cells to specific polarization outcomes in vivo remains limited, and the relative contribution of T-cell-expressed GPR84 versus indirect effects mediated through innate immune cells has not been fully resolved [6]. This represents an important area for future investigation, particularly given the emerging interest in GPR84 as a target in autoimmune diseases where Th17 responses are pathologically amplified.

### Involvement of GPR84 in Neuroinflammatory Disorders

In the central nervous system (CNS), GPR84 is predominantly expressed by microglia and is markedly upregulated in response to diverse neuroinflammatory insults Table 1. Notably, pharmacological activation of GPR84 enhances microglial motility and morphological remodelling without directly inducing pro-inflammatory cytokine expression, distinguishing its signalling profile from that observed in peripheral macrophages and underscoring the context-dependent nature of GPR84 function in the CNS [41]. This complexity is particularly evident in neurodegenerative disorders such as Alzheimer’s disease (AD). GPR84-expressing microglia accumulate in proximity to β-amyloid plaques, where activated microglia contribute to local inflammatory signalling, oxidative stress, and synaptic dysfunction, processes implicated in progressive neurotoxicity [13, 42]. Genetic studies further highlight the complex role of GPR84 in amyloid pathology. In a mouse model of Alzheimer’s disease, GPR84 deficiency reduced microgliosis and microglial accumulation around amyloid plaques but was paradoxically associated with accelerated dendritic degeneration and cognitive decline, suggesting that GPR84-dependent microglial recruitment may exert context-dependent protective effects during early disease stages [12].

**Table 1:** Characteristics of GPR84 agonists and antagonists implicated in neuroinflammatory studies

Compound	Ligand type	Activity	Disease Model	Key Effects	References
Capric acid (C10)	Endogenous	Agonist	Neuroinflammation	Induced microglial motility	[41]
Embelin	Natural	Agonist	Neuroinflammation	Induced microglial motility; Enhanced phagocytosis	[41]
6-OAU	Synthetic	Agonist	AD; Nerve injury	Enhanced microglial motility; Activation of NLRP3 inflammasome	[41]
DIM	Synthetic	Agonist (allosteric)	AD	Decrease in amyloid pathology; promoted microglia recruitment; prevention of cognitive decline	[28]
GLPG1205	Synthetic	Antagonist	Chronic inflammatory disorders (IBD)	Reduced inflammatory response; inhibition of neutrophil migration	[30, 33]
Compound 107	Synthetic	Antagonist	Subarachnoid hemorrhage	Improved neurological deficits; reduced NLRP3 inflammasome formation	[43]

In Parkinson’s disease (PD), direct functional studies linking GPR84 to α-synuclein pathology remain limited. However, given its inducible expression in activated microglia and its role in amplifying innate immune responses, GPR84 is plausibly positioned to participate in inflammatory cascades associated with α-synuclein aggregation and dopaminergic neuronal vulnerability [6, 42]. In multiple sclerosis (MS), transcriptomic and histopathological studies indicate altered microglial activation states within demyelinating lesions, and GPR84 expression has been reported in inflammatory myeloid populations in experimental models. Although mechanistic evidence is still emerging, these findings suggest that GPR84 may contribute to pro-inflammatory polarization in CNS demyelinating conditions [44].

In amyotrophic lateral sclerosis (ALS), microglia progressively adopt neurotoxic inflammatory phenotypes in affected motor regions, correlating with disease severity and neuronal loss [45]. Although direct evidence linking GPR84 to this transition remains limited, its inducible expression in activated microglia suggests a potential contribution to sustained neuroinflammatory states. In models of traumatic CNS injury, GPR84 is rapidly upregulated in CD68⁺ microglia following cortical impact or optic nerve damage, consistent with its role as a microglia-associated receptor expressed under neuroinflammatory conditions [13]. These observations support a broader involvement of GPR84 in injury-associated microglial activation, even though causal mechanisms remain to be fully defined.

Importantly, the role of GPR84 extends beyond neurodegeneration into neuropathic pain. In models of chronic pain, including partial sciatic nerve ligation, Gpr84-deficient mice fail to develop mechanical or thermal hypersensitivity despite preserved macrophage recruitment. These macrophages adopt an anti-inflammatory phenotype, characterized by increased Arg1 and IL-10 expression, reduced production of IL-1β and TNF-α, and elevated intracellular cAMP levels. Similar effects are observed in microglia, where loss of GPR84 signalling attenuates cytokine release and mitigates pain-related behaviours, supporting a central role for GPR84 in shaping neuroimmune mechanisms underlying neuropathic pain [7, 46].

### Therapeutic Potential and Drug Development Targeting GPR84

GPR84 inhibition shows therapeutic promise in preclinical inflammatory and fibrotic models. In DSS-induced colitis, Gpr84-deficient mice exhibit reduced disease severity, intestinal inflammation, and NLRP3 inflammasome activation in macrophages (as detailed in the signalling section) [23]. Pharmacological GPR84 antagonism suppresses inflammatory responses in severe asthma models and limits fibrotic remodelling via effects on macrophages and fibroblasts [32, 47]. In pulmonary and renal fibrosis models, GPR84 suppression reduces macrophage signalling, fibroblast activation, collagen deposition, and TGF-β pathways [33, 47]. Phagocytosis is not a bystander function in the diseases where GLPG1205 failed: in idiopathic pulmonary fibrosis, alveolar macrophage-mediated clearance of apoptotic cells, matrix fragments, and senescent fibroblasts is required to halt and reverse fibrotic remodelling [33, 47], while in Alzheimer's disease, microglial phagocytosis of Aβ plaques, neurofibrillary tangles, and senescent cells is a primary neuroprotective mechanism whose failure accelerates cognitive decline—a conclusion reinforced by the observation that Gpr84-deficient APP/PS1 mice show impaired plaque-associated microglial recruitment alongside accelerated dendritic degeneration and cognitive decline despite reduced microgliosis [12, 48]. Pan-antagonism with GLPG1205 may therefore have inadvertently suppressed the reparative phagocytic programmes required for tissue recovery while leaving redundant cytokine and chemokine pathways insufficiently blocked. The pharmacological separability of GPR84-driven phagocytosis from its NF-κB– and NLRP3-amplifying outputs—demonstrated by G-protein-biased agonists such as DL-175 and OX04528/OX04529, which selectively augment phagocytosis and motility while leaving arrestin-dependent cytokine cascades largely unengaged—argues that next-generation agents should decouple pro-phagocytic from pro-inflammatory GPR84 outputs rather than uniformly suppressing receptor activity, particularly in indications where defective phagocytic clearance is central to pathophysiology [26, 29].

Within the central nervous system (CNS), GPR84 appears to exert context-dependent effects. While pharmacological activation of GPR84 has been shown to promote cytoskeletal remodelling and motility in microglia without directly inducing pro-inflammatory cytokine production in vitro, sustained receptor upregulation in chronic neuroinflammatory settings is associated with reactive glial states and disease progression [4, 12]. In Alzheimer's disease models, GPR84 deficiency reduces microgliosis around amyloid plaques but accelerates dendritic degeneration and cognitive decline. In neuropathic pain, Gpr84 KO mice show reduced hypersensitivity with anti-inflammatory macrophage shifts (increased Arg1, IL-10,reduced IL-1β, TNF-α) via a GPR84–DOK3 axis in microglia [7, 25].

The PINTA trial in idiopathic pulmonary fibrosis did not meet its primary endpoint (FVC change at 26 weeks; *p* = 0.50), although a numerically meaningful treatment difference of 42 mL was observed versus placebo (−34 mL with GLPG1205 vs −76 mL with placebo). However, GLPG1205 demonstrated a poorer safety and tolerability profile than placebo, with higher rates of serious adverse events and treatment discontinuations, most apparent in the nintedanib combination stratum [33]. The ORIGIN trial in ulcerative colitis (NCT02337608) also failed to meet its primary endpoint, despite acceptable tolerability. These translational failures likely reflect a combination of factors: insufficient patient stratification, compensatory activation of parallel inflammatory pathways, and an incomplete understanding of the conditions under which GPR84 signalling is rate-limiting in human disease. Notably, GLPG1205 retains preclinical anti-inflammatory activity in asthma models, suggesting that the translational gap may be indication-specific rather than target-related. Future clinical development of GPR84 antagonists will require improved biomarkers of receptor engagement and more refined patient selection criteria [4, 5, 31–33].

### Current Limitations and Future Directions

Despite a growing body of literature on GPR84 in myeloid cells and the CNS, the field faces several interconnected pharmacological, biological, and translational limitations that substantially constrain mechanistic conclusions. The first concerns the unresolved problem of endogenous ligands of the GPR84 receptor. Although medium-chain fatty acids (C9–C14) and their hydroxylated forms activate GPR84 in vitro, their physiological concentrations in plasma and tissues are generally too low to achieve the potencies required for receptor activation under non-pathological conditions, a problem explicitly acknowledged in the original ligand identification studies [4, 40]. As noted in the cryo-EM structural study by Liu et al. [11], endogenous ligand was assembled into the receptor at an eightfold-above-EC₅₀ concentration throughout complex preparation, underscoring the difficulty of working with endogenous low-affinity MCFAs [11]. GPR84 is therefore still formally considered an orphan receptor, and the identity of the truly relevant endogenous ligand in neuroinflammatory conditions remains unknown [12]. Recently, it was shown that gut dysbiosis-driven elevation of MCFAs activates peripheral myeloid GPR84, triggering neuroinflammation and hippocampal memory deficits, establishing the microbiome as a disease-relevant endogenous ligand reservoir for GPR84 [27].

Following this, the number of tools to work with GPR84 is limited. The species selectivity of these tools further creates a fundamental preclinical barrier for development in the field. The most potent GPR84 antagonists developed to date show marked selectivity for human GPR84 over the mouse orthologue, with affinity differences exceeding 1000-fold in some cases [28]. A consequence is that most preclinical neuroinflammation and pain studies have relied on genetic knockout models, rather than acute pharmacological blockade in adult animals. This raises concerns about developmental compensation and prevents cell-type-specific interpretation. Furthermore, certain widely used agonist tool compounds, most notably PSB-16671, exhibit substantial off-target G-protein activation in primary mouse neutrophils that is preserved in GPR84-knockout animals and is insensitive to GPR84 antagonists – meaning a significant proportion of published "GPR84-mediated" effects in neutrophil studies may be pharmacological artefacts [49].

Much of the future progress in the field will come from the development and preclinical validation of ^1^⁸F-labelled GPR84 PET tracers, that show high specificity and affinity to human GPR84-expressing cells and detect neuroinflammation in vivo in LPS-challenged and 5xFAD mice [50]. Because GPR84 is selectively upregulated only on pro-inflammatory myeloid cells, those biomarkers offer the first imaging tool capable of specifically tracking activated microglia and macrophages in vivo, with stated goals of clinical translation for disease staging and therapeutic monitoring in neurodegeneration.

Another significant roadblock in the studies of the role of GPR84 in neuroinflammation is conflicting findings across biological contexts. The central unresolved contradiction in the GPR84 literature is whether the receptor is predominantly pro- or anti-inflammatory. In LPS-activated macrophages, GPR84 agonism amplifies IL-12, p40, TNF-α, and CCL2 via NF-κB/Akt/ERK, supporting a pro-inflammatory role [22, 40]. Yet despite consistently elevated GPR84 expression in APP/PS1 Alzheimer’s disease microglia, full GPR84 deletion paradoxically accelerated cognitive decline and dendritic degeneration without affecting plaque load and completely failed to influence clinical progression in EAE or endotoxic shock [12]. These studies encapsulate the central therapeutic challenge posed by GPR84: its pro-phagocytic and pro-inflammatory outputs are not only pharmacologically separable but also temporally dissociated across disease progression. In acute neuroinflammatory injury, GPR84 antagonism is beneficial, yet in chronic neurodegeneration the same receptor sustains the microglial recruitment and phagocytic priming required for plaque compaction, neuropil maintenance, and debris clearance. Sustained, non-selective antagonism is therefore an intrinsically limited strategy for chronic neurodegeneration: it sacrifices reparative phagocytic capacity while providing only partial and time-limited suppression of an inflammatory tone maintained by redundant pathways. It was shown in this review that GPR84 is not a uniformly pro-inflammatory receptor and that its function is profoundly context-dependent—a variable that most mechanistic studies, which focus on a single model or stimulus, are not designed to address. It might be that GPR84 acts as a stage-dependent switch in chronic neuroinflammations, though to obtain a definitive answer, the field needs to implement multiomics, spatial, and temporal techniques.

Since microglia have been at the centre of neuroinflammation research for decades, it is particularly concerning for GPR84 studies that direct mechanistic evidence in microglia remains disconnected from behavioural outcomes. Studies showed that GPR84 agonists promote microglial motility and cytoskeletal reorganisation via Gαi/o but fail to drive NF-κB nuclear translocation or induce pro-inflammatory cytokine transcription (IL-1β, IL-6, TNF-α) in isolated microglia [41]. This dissociation between a well-established phagocytic/migratory response and direct cytokine induction means that the inflammatory output attributed to GPR84 in complex models may reflect indirect consequences, like microglia recruitment facilitating subsequent cytokine release, rather than a direct transcriptional programme. Consistently, Nicol et al. [7] demonstrated that microglial Iba1 and phospho-p38 MAPK responses in the spinal cord were entirely unaffected by GPR84 deletion in neuropathic pain models,the behavioural protection was attributable to peripheral macrophage reprogramming [7]. This microglia versus infiltrating macrophages ambiguity runs through GPR84 literature and would have to be systematically resolved with the use of conditional knockouts.

Last, but not least, the clinical translation record for GPR84 is, at best, inconclusive. GLPG1205 failed to meet its primary endpoint both in the Phase IIa ORIGIN trial and Phase II PINTA trial. On top of that, GLPG1205 demonstrated a worse safety and tolerability profile than placebo, with higher rates of serious adverse events and treatment discontinuations [33]. While these failures do not disprove GPR84 as a target, they fundamentally undercut the assumption that preclinical macrophage GPR84 biology will translate straightforwardly to clinical benefit.

## Conclusions

GPR84 should be understood not as a pro-inflammatory on–off switch, but as a regulatory node embedded in a broader immunometabolic network, one whose signalling output is fundamentally determined by context rather than intrinsic receptor identity. This distinction matters because the same receptor manipulation that attenuates neuropathic pain and bowel inflammation in one setting can, in another, strip microglia of the recruitment capacity they need to protect neurons from amyloid-driven degeneration. The double-edged nature of GPR84 manipulation is not an exception to its biology – it is the defining feature of it, and the field has been slow to treat it as such. Interpreting GPR84 as predominantly pro-inflammatory, and therapeutic inhibition as straightforwardly beneficial, is likely too simplistic a framework to yield durable clinical success.

The more uncomfortable possibility, supported by the pattern of inconclusive and sometimes contradictory results, is that the field is not yet studying GPR84 in a way that faithfully reflects its biology. When most mechanistic insights are derived from isolated myeloid cells stimulated with synthetic ligands at concentrations that may never occur in human tissue, when constitutive knockout models conflate microglial and peripheral macrophage contributions across developmental time, and when the primary pharmacological antagonists available are essentially non-functional at the murine receptor, the data generated will systematically misrepresent what the receptor actually does in a living organism with disease. The clinical failures of GLPG1205 can be read not as evidence that GPR84 is a poor target, but as evidence that preclinical models built on these methodological foundations were insufficient to predict the complexity of human GPR84 engagement.

Until the field develops tools appropriate for GPR84 biological complexity – conditional, cell-type-specific genetic models that can isolate microglial from peripheral myeloid contributions; CNS-penetrant, species-agnostic antagonists that model human receptor pharmacology in murine disease; validated in vivo biomarkers capable of confirming target occupancy; and biased ligands that can separate phagocytic and migratory GPR84 outputs from its pro-inflammatory cytokine-amplifying effects – the risk of drawing the wrong therapeutic conclusions will persist. Critically, single-readout and endpoint-only experimental designs are fundamentally mismatched to a receptor whose function is defined by context. GPR84 expression, signalling output, and immune consequence all vary across cell subtypes within the same tissue, shift dynamically across disease stages, and are shaped by the local metabolic and cytokine microenvironment in ways that bulk transcriptomics and population-level assays cannot resolve. Integrating spatial transcriptomics and spatial proteomics to map GPR84-expressing myeloid subpopulations at cellular resolution within intact CNS tissue will be essential to determine not merely whether GPR84 is expressed, but in which cells, at which disease stage, under which ligand conditions, and with which downstream consequences. Without this dimensional resolution, the field will continue to generate findings that are locally reproducible but globally irreconcilable. Promising targets have been abandoned, and potentially effective interventions have likely failed in trials, not because the biology was intractable, but because it was interrogated with instruments too blunt to resolve the spatial, temporal, and cell-type-specific context-dependence that defines it. GPR84 may yet prove to be a therapeutically meaningful aim in neuroinflammation but realising that potential will require the field to first acknowledge that it has been looking at GPR84 through a lens that flattens the very complexity that makes the receptor worth targeting.

## Data Availability

No datasets were generated or analysed during the current study.
